# Vacuum Assisted Closure- utilization as home based therapy in the management of complex diabetic extremity wounds

**DOI:** 10.12669/pjms.311.6093

**Published:** 2015

**Authors:** Kamran Hafeez, Haroon-ur-Rashid, Ghulam Mustafa Kaim Khani, Darshan Kumar, Sunil Kumar

**Affiliations:** 1 Dr. Kamran Hafeez, FCPS, Assistant Professor Orthopedics, Dow International Medical College, Dow University of Health Sciences, Ojha Campus Suparco Road, Karachi, Pakistan.; 2 Dr. Haroon-ur-Rashid, FCPS, Assistant Professor Orthopedics, Aga Khan University Hospital, Stadium Road, Karachi, Pakistan.; 3 Dr. Ghulam Mustafa Kaim Khani, FCPS, Associate Professor Orthopedics, Dow International Medical College, Dow University of Health Sciences, Ojha Campus Suparco Road, Karachi, Pakistan.; 4 Dr. Darshan Kumar, FCPS, Assistant Professor Medicine, Dow International Medical College, Dow University of Health Sciences, Ojha Campus Suparco Road, Karachi, Pakistan.; 5 Dr. Sunil Kumar, FCPS, Assistant Professor Orthopedics, Dow International Medical College, Dow University of Health Sciences, Ojha Campus Suparco Road, Karachi, Pakistan.

**Keywords:** Diabetic wound, Vacuum assisted closure, Complex lower limb wounds

## Abstract

**Objective:**

Vacuum assisted closure is a reported technique to manage complex wounds. We have utilized this technique by using simple locally available material in the management of our patients on outpatient basis. The objective of this study is to present our experience.

**Methods:**

This study was conducted from June 2011 to June 2013 at Dow University Hospital and Aga Khan University Hospital, Karachi. There were 38 patients managed with vacuum assisted closure. Mean age was 56±7.8 years. Twenty three patients presented with necrotizing fasciitis and 15 patients with gangrene. Lower limbs were involved in majority of the patients. Debridement or amputations were done. Vacuum dressing was changed twice weekly in outpatient department. Wounds were closed secondarily if possible or covered with split thickness skin graft in another admission.

**Results:**

All the wounds were successfully granulated at the end of vacuum therapy. Mean hospital stay was 7.5 days. Vacuum dressing was applied for a mean of 20 days. There was reduction in the size of the wound. Thirteen patients underwent secondary closure of the wound under local anesthesia, 18 patients required coverage with split thickness skin graft and 7 patients healed with secondary intention.

**Conclusion:**

Vacuum assisted closure appeared to be an effective method to manage complex diabetic wounds requiring sterile wound environment.

## INTRODUCTION

Diabetes Mellitus is a complex disease resulting in number of complications including diabetic foot ulcers.^1^ Lifetime risk of developing foot ulcers in diabetic patients is reported from 15% to as high as 25% in literature.^2^ Failure to manage these wounds in an efficient manner may lead to limb amputation which is the most disastrous outcome of these ulcers.^3^^,^^4^ Peripheral neuropathy and abnormal pressure distribution lead to development of these ulcers.^5^ Poor blood supply, deranged intrinsic wound healing capacity^6^ and impaired immunity make these wounds difficult to manage.^7^ These wounds take a lot of time to heal and require daily dressing in a sterile environment in order to prevent secondary infection. Continuous management within hospital will definitely increase the treatment cost.

 Different dressing techniques and materials have been utilized in the management of these complex wounds. Vacuum assisted closure is an effective technique which keeps the wound environment sterile and does not require frequent change of dressings. It has been claimed to reduce the wound size by contracting the wound margins and enhances local vascularity. The rate of granulation formation in the wound bed is also enhanced through negative pressure wound therapy.^8^ Commercially available negative pressure vacuum apparatus^9^ is not available in our setting, so we have utilized simple readily available material to manage these wounds with negative pressure therapy on outpatient basis. The objective of this study was to share our experience with this technique.

## METHODS

This retrospective audit was conducted from April 2011 to June 2013 at Dow University Hospital and Aga Khan University Hospital, Karachi. All the diabetic patients admitted with extremity wounds complicated with gangrene or necrotising infections who underwent some surgical intervention (debridement/amputation) followed by dressing with vacuum assisted closure technique were included in the study. There were 38 patients managed with vacuum assisted closure. All the patients were diabetic while 18 had other co-morbids like hypertension and ischemic heart disease. Twenty three patients presented with necrotizing fasciitis and 15 patients with gangrene. Lower limbs were involved in majority of the patients (foot in 16 patients and leg in 18 patients) having wounds with exposed tendons, fascia or bone. Two patients had involvement of forearm and two over arm. Debridement was done in 18 patients, below knee amputation in 11 patients, ray amputation in 8 patients and transmetatarsal amputation in one patient. Vacuum dressing was applied in all the cases.

**Fig.1:**
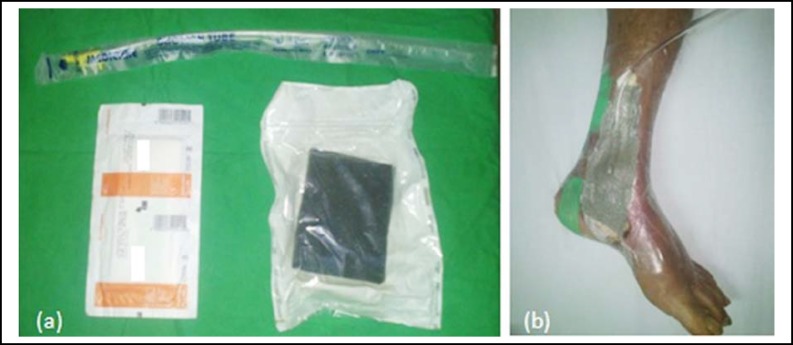
Technique.

***Technique:*** After debridement and excision of all necrotic tissue, a piece of sterilized sponge foam is cut according to the size of the wound and placed in the wound over a piece of gauze. Suction catheter (size 18 F or above) was positioned in between the two layers of foam. Adhesive transparent dressing was used to seal the wound (Fig.1). Suction catheter was then attached to either central suction system available in the ward or to the suction machine. Patient was taught to connect the tube with the machine and to maintain the pressure. Negative pressure of -50 to -100 mmHg was maintained.

Patients were discharged as soon as their medical condition was stable. Machine was arranged on rental basis for home therapy. Patients were followed in the outpatient department and vacuum dressing was changed twice weekly. Wound dimensions were recorded both at the beginning and at the end vacuum dressing. Wounds were closed secondarily if possible under local anesthesia, allowed to heal with secondary intention or covered with split thickness skin graft in another admission.

## RESULTS

Thirty eight patients were managed with this technique. Mean age was 56 +/- 7.8 years. There were 31 male and 7 female patients. Mean hospital stay was 7.5 days (range 3-23 days). After surgery average width of the wound was 7.5 cm (range 3-14 cm) and length of the wound was 11.5 cm (range 6-20 cm) before application of vacuum dressing. Vacuum dressing was applied for a mean of 20 days (range 12 – 70 days). All the wounds responded to the vacuum therapy and wound dimensions were reduced. At the end of vacuum therapy the average width of the wound was 5.7 cm (range 1-10 cm) and the length was 9 cm (range 3-16 cm). Seven patients required additional surgical debridements during the course of their treatment. All the wounds were successfully granulated at the end of vacuum therapy. Thirteen patients underwent secondary closure of the wound under local anesthesia, 18 patients required coverage with split thickness skin graft and 7 patients healed with secondary intention. There was no treatment related complication.

**Fig.2:**
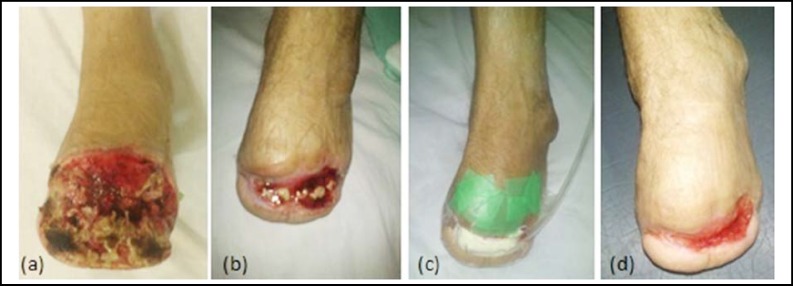
Forty year old male with transmetatarsal amputation.

***Case-1: ***Forty year old male known diabetic presented with gangrene of toes, underwent transmetatarsal amputation. His wound got infected and failed to heal with routine dressings. Debridement was done and vacuum dressing was applied. At the end of 3 months wound contracted and healed with secondary intention (Fig.2).

**Fig.3:**
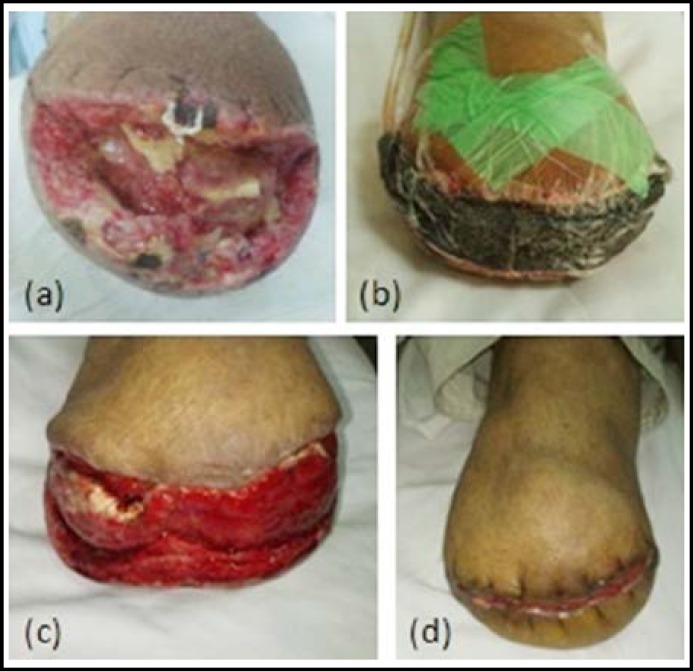
Fifty six year old male with below knee amputation.

***Case-2: ***Fifty six year old male presented with infected below knee amputation stump. Debridement was done and the wound margins were freshened. Vacuum dressing was applied for 12 days and wound was closed with sutures under local anesthesia (Fig.3).

## DISCUSSION

In our study all the wounds granulated successfully at the end of vacuum dressings. There was decrease in dimensions of the wounds after completion of treatment with seven wounds healed with secondary intention not requiring secondary suturing or skin graft.

Negative pressure vacuum dressing have been utilized in the management of complex wounds.^10^^,^^11^ Continuous or intermittent suction keep the wound clear of exudate, persistence of which may be a good medium for bacterial growth and thus decreases the bacterial count. It also enhances the blood supply and proliferation of granulation tissue.^12^ Moues CM et al.^13^ reviewed 400 peer reviewed studies related to topical negative pressure therapy and concluded that it helps in reducing wound size, promote angiogenesis and increase blood flow to the wound site. However decrease in bacterial count and edema were not proven in their research. Commercially available vacuum assisted closure devices are not currently available in our setting. These machines are designed to provide alternating cycles of negative pressure at the wound site. We have utilized the negative pressure therapy dressing with the locally available dressing material and connected with continuous suction while admitted in the ward. After discharge intermittent pressure was used but at longer intervals (2 hours on negative pressure while half an hour without suction). Negative pressure therapy has an added advantage of less frequent dressings which may be associated with pain in case of large wounds. Occlusive nature of the dressing kept the wound sterile and enhances granulation formation.^14^ In our study dressing was changed twice weekly.

Ghani U et al.^15^ utilized the similar method of negative pressure therapy with intermittent application of pressure in 52 patients mainly in traumatic wounds. They noticed 68% reduction in the size of wound at the end of therapy and healthy granulation tissue formation in majority of the wounds. 

Iqbal MZ et al.^16^ utilized this technique in management of 25 patients with non healing wounds, diabetes as a cause in majority of patients. They have utilized continuous suction in 40% of the patients and intermittent suction after every 2 hours in 60% of the patients. All of their patients responded well to vacuum therapy. Four patients healed with secondary intention, five required secondary suturing and remaining required skin graft. In our study all the patients were diabetic with additional co morbid conditions in some patients. All wounds had good granulation tissue. Seven healed with secondary intention, 13 required secondary suturing and 18 required skin graft.

Negative wound therapy is considered to be superior to conventional treatment modalities.^17^ Blume PA et al.^18^ compared vacuum assisted closure with advanced moist wound therapy in a multicenter randomized control trial. They reported results in 342 patients. A greater proportion of patients managed with vacuum assisted closure (43.2%) achieved complete ulcer closure as compared to the moist therapy (28.9%). They concluded it to be safe and efficacious as compared to advanced moist therapy. Unfortunately in our study there was no comparison group. We achieved complete closure of the wound in seven patients (18.4%). Baharestani MM et al.^19^ compared early versus late initiation of negative pressure therapy utilized in the management of stage III or IV pressure ulcers and surgical wounds and their effect on length of stay in home health care. Early initiation was associated with shorter stay.

Limited number of patients, retrospective nature of this report and non availability of comparison group were the limitations of our study. Management of these complex wounds on outpatient basis reduces the hospital stay which in turn reduces the cost of treatment making it also a cost effective option in addition to its other advantages.

## CONCLUSION

Vacuum assisted closure appeared to be an effective method to manage complex diabetic wounds requiring sterile wound environment. Vacuum therapy made it possible to keep the wound sterile, free of exudates by continuous suction and helped in granulation for all wounds thus making it possible to close them by secondary intention, by secondary suturing or by skin graft. Application of vacuum therapy on outpatient basis also made it possible to decrease the hospital stay.

**(a)** Suction catheter, adhesive transparent dressing and sterilized sponge foam.

**(b)** Vacuum dressing applied over leg of a patient. Sterilized sponge foam with a suction catheter in between is visible and sealed with adhesive transparent dressing.

**(a)** Necrotic slough in wound. **(b)** Wound size decreased with little amount of slough.

**(c) **Vacuum dressing applied. **(d)** Wound contracted and near to heal.

**(a)** Infected stump. **(b)** Vacuum dressing applied after debridement.

**(c)** Healthy wound. **(d)** Secondary closure.

## Authors’ contribution:

**KH:** Conception and design, drafting of article.

**HR:** Drafting and revision of article, final approval.

**GMKK:** Interpretation of data, drafting and revision of article.

**DK and SK:** Acquisition of data and analysis.
